# Novel α-MSH Peptide Analogues with Broad Spectrum Antimicrobial Activity

**DOI:** 10.1371/journal.pone.0061614

**Published:** 2013-04-23

**Authors:** Paolo Grieco, Alfonso Carotenuto, Luigia Auriemma, Antonio Limatola, Salvatore Di Maro, Francesco Merlino, Maria Luisa Mangoni, Vincenzo Luca, Antonio Di Grazia, Stefano Gatti, Pietro Campiglia, Isabel Gomez-Monterrey, Ettore Novellino, Anna Catania

**Affiliations:** 1 Department of Pharmacy, University of Naples Federico II, Naples, Italy; 2 Istituto Pasteur-Fondazione Cenci Bolognetti, Department of Biochemical Science, ‘A. Rossi Fanelli’, University of Rome ‘La Sapienza’, Rome, Italy; 3 Center for Preclinical Investigation, Fondazione IRCCS Ca'Granda - Ospedale Maggiore Policlinico, Milano, Italy; 4 Department of Pharmaceutical Science, University of Salerno, Fisciano, Salerno, Italy; The Scripps Research Institute and Sorrento Therapeutics, Inc., United States of America

## Abstract

Previous investigations indicate that α-melanocyte-stimulating hormone (α-MSH) and certain synthetic analogues of it exert antimicrobial effects against bacteria and yeasts. However, these molecules have weak activity in standard microbiology conditions and this hampers a realistic clinical use. The aim in the present study was to identify novel peptides with broad-spectrum antimicrobial activity in growth medium. To this purpose, the Gly10 residue in the [*D*Nal(2′)-7, Phe-12]-MSH(6–13) sequence was replaced with conventional and unconventional amino acids with different degrees of conformational rigidity. Two derivatives in which Gly10 was replaced by the residues Aic and Cha, respectively, had substantial activity against Candida strains, including *C. albicans*, *C. glabrata*, and *C. krusei* and against gram-positive and gram-negative bacteria. Conformational analysis indicated that the helical structure along residues 8–13 is a key factor in antimicrobial activity. Synthetic analogues of α-MSH can be valuable agents to treat infections in humans. The structural preferences associated with antimicrobial activity identified in this research can help further development of synthetic melanocortins with enhanced biological activity.

## Introduction

α-Melanocyte stimulating hormone (α-MSH), a pro-opiomelanocortin (POMC) derivative, is an ancient tridecapeptide that exerts pleiotropic influences on the host physiology [1]. A major contribution of α-MSH to tissue protection resides in its capacity to prevent cell injury induced by harmful stimuli including endotoxin, reperfusion injury, blood loss, and oxidative stress [1], [2]. Of interest, α-MSH involvement in host defense includes antimicrobial activity [3], [4]. Indeed, the peptide and its C-terminal sequence Lys-Pro-Val were found to inhibit growth of both the yeast *Candida albicans* and the gram-positive bacterium *Staphylococcus aureus* [4]; further, the N-terminal sequence His-Phe-Arg-Trp showed antimicrobial activity against *Cryptococcus neoformans* [5].

Although the natural α-MSH peptide has a very short half-life that makes it unsuitable for clinical use, synthetic analogues of it could form the basis for novel antimicrobial agents. However, key issues need to be solved before a therapeutic use is realistic. A crucial question concerns potency of the antimicrobial activity. Indeed, α-MSH and the synthetic derivatives described to date were found to exert their activity against infectious agents that were suspended in physiologic solution or in water but not in culture media that allow microorganisms to grow [4], [6], [7], [8], [9], [10]. Therefore, although data indicate a potential for melanocortin derivatives to combat infections, none of the known molecules is active against microorganisms in growth medium. Because this is a major obstacle to a realistic clinical use, the aim in this research was to design novel melanocortin analogues that could overwhelm this weakness. The lead sequence selected was [*D*Nal(2′)-7, Phe-12]–MSH(6–13) (*DNal*), a promising antimicrobial compound that contains the invariant ‘*core*’ sequence His-Phe-Arg-Trp (6–9) common to all melanocortins [7]. Indeed, although *DNal* did not kill Candida in growth medium, it did kill 100% organisms incubated in distilled water [7]. Conformational analysis of *DNal* indicated the presence of two β-turns along residues 7–10 and 10–13 (type I, and distorted type III, respectively) [11]; these two turns, which likely form the pharmacophoric moieties, are linked by a highly flexible glycine residue that can orient them differently [11]. Therefore, in the present research we produced novel antimicrobial peptides based on replacement of Gly10 in the *DNal* sequence with natural and unnatural amino acids with different degrees of conformational rigidity (Table 1). Activity against various pathogenic agents including gram-positive and gram-negative bacteria and yeasts was assessed using standard culture tests. We report results of a conformational study on the most effective peptides.

**Table 1:** Lead sequence *DNal* and Gly10 substituted peptides.

Peptide	Sequence
***DNal***	H-His-*D*(2′)Nal-Arg-Trp-**Gly**-Lys-Phe-Val-NH_2_
**1**	H-His-*D*(2′)Nal-Arg-Trp-**Aib**-Lys-Phe-Val-NH_2_
**2**	H-His-*D*(2′)Nal-Arg-Trp-**Deg**-Lys-Phe-Val-NH_2_
**3**	H-His-*D*(2′)Nal-Arg-Trp-**tBuGly**-Lys-Phe-Val-NH_2_
**4**	H-His-*D*(2′)Nal-Arg-Trp-**Ac3c**-Lys-Phe-Val-NH_2_
**5**	H-His-*D*(2′)Nal-Arg-Trp-**Ac4c** -Lys-Phe-Val-NH_2_
**6**	H-His-*D*(2′)Nal-Arg-Trp-**Ac5c**-Lys-Phe-Val-NH_2_
**7**	H-His-*D*(2′)Nal-Arg-Trp-**Ac6c** -Lys-Phe-Val-NH_2_
**8**	H-His-*D*(2′)Nal-Arg-Trp-**Aic**-Lys-Phe-Val-NH_2_
**9**	H-His-*D*(2′)Nal-Arg-Trp-**Phe**-Lys-Phe-Val-NH_2_
**10**	H-His-*D*(2′)Nal-Arg-Trp-**Cha**-Lys-Phe-Val-NH_2_
**11**	H-His-*D*(2′)Nal-Arg-Trp-**βAla**-Lys-Phe-Val-NH_2_
**12**	H-His-*D*(2′)Nal-Arg-Trp-**Acpc**-Lys-Phe-Val-NH_2_
**13**	H-His-*D*(2′)Nal-Arg-Trp-**Gly-Gly**-Lys-Phe-Val-NH_2_

## Materials and Methods

### Materials

*N*9-fluorenylmethoxycarbonyl (Fmoc)-protected natural amino acids were purchased from GLS Biochem (Shangai-China), *N*Fmoc-protected special amino acids from NeoMPS (Stasbourg-France) and Chem-Impex International, Inc. (Wood Dale-Illinois), 2-(1*H*-benzotriazole-1-yl)-1,1,3,3-tetramethyluroniumhexafluoro- phosphate (HBTU) and *N*-hydroxybenzotriazole (HOBt) from Iris Biotech GmbH (Marktredwitz-Germany) and Rink amide resin from Advanced Chemtech (Lousville-KY). For the *N*–Fmoc-protected amino acids, the following side chain protecting groups were used: Arg(*N* ^γ^-2,2,4,6,7-pentamethyl-dihydrobenzofuran-5-sulfonyl (Pbf)), His(*N* ^im^- triphenylmethyl(trityl) (Trt)), Trp(*N* ^in^- *tert*-butyloxycarbonyl (Boc)), Lys(Boc). Peptide synthesis solvents, reagents, as well as CH_3_CN for HPLC were reagent grade and were acquired from commercial sources and used without further purification unless otherwise noted. ESI-MS analyses were performed by API 2000. The purity of the finished peptides was checked by analytical reversed-phase high performance liquid chromatography (RP-HPLC) using a Shimadzu mod. CL-10ADVP system with a built-in diode array detector. In all cases, the purity of the finished peptides was greater than 95% as determined by these methods.

### General Method for Peptide Synthesis and Purification

All peptides were synthesized using the solid-phase method of peptide synthesis and purified by RP-HPLC. Each peptide was synthesized on 0.250 g of Rink amide resin (substitution 0.75 mmol/g) by manual method using *N*-Fmoc chemistry and an orthogonal side chain protection strategy. The resin was first swollen in dichloromethane (DCM)/(*N,N*-dimethylformamide) DMF (1∶1) for 2 h and the following amino acids were then added to the growing peptide chain by stepwise addition of *N*-Fmoc-Val-OH, *N*-Fmoc-Phe-OH, *N*-Fmoc-Lys(Boc)-OH, *N*-Fmoc-(L)-AA-OH (AA: Gly-Gly dipeptide, β-alanine, β-cyclohexylalanine, 1-amino-cyclopentane carboxylic acid, 1-amino-cyclopropane carboxylic acid, α-t-butylglycine, 2-aminoindane-2-carboxylic acid, 1-amino-cyclobutane carboxylic acid, (1R,2R)-2-aminocyclopentane-1-carboxylic acid, diethylglycine, 1-amino-cyclohexanecarboxylic acid, phenylalanine; Figure S1), *N*-Fmoc-Trp(Boc)-OH, *N*-Fmoc-Arg(*N^γ^*-Pbf)-OH, *N*-Fmoc-(D)-2-Nal-OH, and *N*-Fmoc-His(*N* ^im^-Trt)-OH, using standard solid phase methods. Each coupling reaction was achieved using a 3-fold excess of each amino acid, HBTU, and HOBt in presence of a 6-fold excess of (*N,N*-diisopropylethylamine) DIPEA for 1 h. Deprotection of the *N*-Fmoc group was carried out by treating the protected peptide resin with 25% piperidine solution in DMF (1×4 mL, 20 min). After each coupling and deprotection, the peptide resin was washed with DMF (3×4 mL), DCM (3×50 mL) and again with DMF. The peptide sequences were thereafter assembled by alternate cycles of coupling and deprotection. After coupling of the *N*-terminal amino acid, the *N*-terminal Fmoc group was deblocked as described above and the peptide-resin was thoroughly washed with DCM (4×25 mL) and dried under argon to yield dried peptide-resin.

The peptide-resin was then cleaved by treatment with 5 mL of a solution of triethylsilane (Et_3_SiH) (5%), water (5%), in trifluoroacetic (TFA) with shaking at room temperature for 3 h. The resin was then removed from the solution by filtration and the crude peptide was recovered by precipitation with cold anhydrous ethyl ether. Centrifugation at 1500 *g* for 3 min followed by decantation of the supernatant ether and air-drying of the residue yielded the crude peptide as a white to pale beige-colored amorphous solid.

Final peptide purification was achieved using a preparative RP-HPLC Phenomenex Jupiter Proteo, 10 m 90 Å. The peptide samples were injected onto the column at a concentration of 20 mg/mL in 20% aqueous CH_3_CN and were eluted with a CH_3_CN gradient (10 to 90%) over 40 min at a flow rate of 15.0 mL/min, with a constant concentration of TFA (0.1% v/v). The separations were monitored at 230 and 254 nm and integrated with a Shimadzu diode array detector mod. SPD-M10AVP dual wavelength absorbance detector model UV-D. Fractions corresponding to the major peak were collected, pooled, and lyophilized to yield the final peptides as pure (>95%) white solids. The final yields of the peptides ranged between 42 and 57%. The analytical data and the amino acid analysis for each compound are reported in Tables S1, S2 and Figure S2.

### Candida spp

Two *Candida albicans* isolates were purchased from the ATCC (No. 24433 and 76615). The other yeast isolates were obtained from the collection of Fondazione Ca' Granda- Ospedale Maggiore Policlinico, Milano. The collection included *C. albicans* (7 isolates), *C. glabrata* (3 isolates), and *C. krusei* (3 isolates).

### Bacteria

The strains used for the antimicrobial assays were the Gram-negative bacteria *Acinetobacter baumani* ATCC 19606, *Escherichia coli* D21, *Pseudomonas aeruginosa* ATCC 27853, and *Pseudomonas syringae* pv tabaci 1918NCPPB and the Gram-positive bacteria *Bacillus megaterium* Bm11, *Staphylococcus aureus* ATCC 25923, and *Staphylococcus epidermidis* ATCC 12228.

### Anti-Candida assay

Antifungal susceptibility testing was performed using the broth microdilution method according to the NCCLS M27-A guidelines (National Committee for Clinical Laboratory Standards. 1997, Reference method for broth dilution antifungal susceptibility testing of yeasts and approved standard NCCLS document M27-A. National Committee for Clinical Laboratory Standards, Wayne, Pa.). The organisms were removed from frozen glycerol stock (10% sterile glycerol suspensions stored at −70°C), subcultured onto Sabouraud's dextrose plates, and incubated at 35°C. After 48 h of incubation, the plates were inspected for purity. A colony was taken from the agar plate and transferred into 5 mL Sabouraud-dextrose broth and incubated for 48 h at 35°C. Cells were centrifuged at 1,000× g for 10 min and the pellet was washed twice with distilled water. Cells were counted and suspended in RPMI 1640 medium buffered to pH 7.0 with 0.165 mol l^−1^ morpholinepropanesulphonic acid (MOPS) buffer (Sigma) to obtain the two times test inoculum (1×10^3^ to 5×10^3^ CFU/mL). Each well of 96 U-shaped well-plates received 100 µl of each antifungal peptide in concentrations from 10^−4^ to 7.8×10 ^−7^ M.

The plates were incubated at 35 C and were observed for growth at 48 h. The MIC90, i.e. the minimum inhibitory concentrations endpoint were determined as 90% reduction in turbidity measured using a spectrophotometer (Titertek multiscan at 690 nm wave length).

### Antibacterial assay

Susceptibility testing was performed by adapting the microbroth dilution method outlined by the Clinical and Laboratory Standards Institute, using sterile 96-well plates (Falcon NJ, USA). The bacterial growth was aseptically measured by absorbance at 590 nm with a spectrophotometer (UV-1700 Pharma Spec Shimadzu, Tokyo, Japan). Afterwards, aliquots (50 µl) of bacteria in mid-log phase at a concentration of 2×10^6^ colony-forming units (CFU)/mL in culture medium (Mueller-Hinton, MH) were added to 50 µl of MH broth containing the peptide in serial 2-fold dilutions ranging from 1.56 to 100 µM. Inhibition of microbial growth was determined by measuring the absorbance at 590 nm, after an incubation of 18 h at 37°C with a microplate reader (Infinite M200; Tecan, Salzburg, Austria). Antibacterial activity was expressed as the minimal inhibitory concentration (MIC), the concentration of peptide at which 100% inhibition of microbial growth is observed after 18 h of incubation.

### Hemolytic assay

The hemolytic activity was measured on human red blood cells as reported previously [12]. Briefly, aliquots of a human erythrocyte suspension in 0.9% (w/v) NaCl were incubated with serial two-fold dilutions of peptide (dissolved in 20% ethanol prior to use) for 40 min at 37°C with gentle mixing. The samples were then centrifuged for 5 min at 900 g and the absorbance of the supernatant was measured at 415 nm. Complete lysis was measured by suspending erythrocytes in distilled water [13].

### Cytotoxic activity

Effects on viability was tested in the immortalised keratinocyte cell line HaCaT using the [3(4,5-dimethylthiazol-2yl)2,5-diphenyltetrazolium bromide] (MTT) colorimetric method in which the intensity of the dye is proportional to the number of viable cells. MTT is a tetrazolium salt which is reduced to a colored formazan product by mitochondrial reductases present only in metabolically active cells. Cells were cultured in Dulbecco's modified Eagle's medium (DMEM; Sigma) supplemented with 10% heat inactivated fetal bovine serum, glutamine (4 mM) and gentamicin. Afterwards, cells were plated in triplicate wells of a microtiter plate, at 4×10^4^ cells/well in DMEM supplemented with 2% serum without antibiotic. After overnight incubation at 37°C in a 5% CO_2_ atmosphere, the medium was replaced with 100 l fresh DMEM supplemented with the peptides at different concentrations. The plate was incubated for 24 h at 37°C in a 5% CO_2_ atmosphere. Then, DMEM was removed and replaced with Hank's medium (136 mM NaCl; 4.2 mM Na_2_HPO_4_; 4.4 mM KH_2_PO_4_; 5.4 mM KCl; 4.1 mM NaHCO_3_, pH 7.2, supplemented with 20 mM D-glucose) containing 0.5 mg/ml MTT. After 4 h incubation, the formazan crystals were dissolved by adding 100 l of acidified isopropanol and absorption of each well was measured using a microplate reader (Infinite M200; Tecan, Salzburg, Austria) at 570 nm. Cell viability was e expressed as percentage of viability in control cells (cells not treated with peptide).

### Nuclear Magnetic Resonance (NMR) spectroscopy

The samples for NMR spectroscopy were prepared by dissolving the appropriate amount of peptide in 0.55 mL of ^1^H_2_O, 0.05 mL of ^2^H_2_O and 160/40 mM dodecylphosphocholine (DPC) -d_38_/sodium dodecylsulphate (SDS)-d_25_ micelles solution to obtain a concentration of 1–2 mM peptide. The NMR experiments were performed at pH 5.0. NH exchange studies were made by dissolving peptide in 0.60 mL of ^2^H_2_O and 200 mM 160/40 mM DPC-d_38_/SDS-d_25_. NMR spectra were recorded on a Varian Unity INOVA 700 MHz spectrometer equipped with a z-gradient 5 mm triple-resonance probe head. All the spectra were recorded at a temperature of 25°C. The spectra were calibrated relative to 3-(trimethylsilanyl)propionic acid (0.00 ppm) as internal standard. One-dimensional (1D) NMR spectra were recorded in the Fourier mode with quadrature detection. 2D double quantum filtered correlated spectroscopy (DQF-COSY) [14], total correlation spectroscopy (TOCSY) [15], and nuclear Overhauser enhancement spectroscopy (NOESY) [16] spectra were recorded in the phase-sensitive mode using the method from States [17]. Data block sizes were 2048 addresses in t_2_ and 512 equidistant t_1_ values. A mixing time of 70 ms was used for the TOCSY experiments. NOESY experiments were run with mixing times in the range of 150–300 ms. The water signal was suppressed by gradient echo [18]. The 2D NMR spectra were processed using the NMRPipe package [19]. Before Fourier transformation, the time domain data matrices were multiplied by shifted sin^2^ functions in both dimensions, and the free induction decay size was doubled in F1 and F2 by zero filling. The qualitative and quantitative analysis of DQF-COSY, TOCSY and NOESY spectra were obtained using the interactive program package XEASY [20]. *J*_HN-Hα_ couplings were difficult to measure probably because of a combination of small coupling constants and broad lines. The temperature coefficients of the amide proton chemical shifts were calculated from 1D ^1^H NMR and 2D TOCSY experiments performed at different temperatures in the range 298–320 K by means of linear regression.

### Structural determinations

The NOE-based distance restraints were obtained from NOESY spectra collected with a mixing time of 200 ms. Peak volumes were translated into upper distance bounds with the CALIBA routine from the DYANA software package [21]. The requisite pseudoatom corrections were applied for non-stereospecifically assigned protons at prochiral centers and for the methyl group. After discarding redundant and duplicated constraints, the final list of constraints was used to generate an ensemble of 200 structures by the standard DYANA protocol of simulated annealing in torsion angle space. No dihedral angle restraints and no hydrogen bond restraints were applied. An error tolerant target function (tf type 3) was used to account for the peptide intrinsic flexibility. Then, 20/200 structures were chosen, whose interproton distances best fitted NOE derived distances, and refined through successive steps of restrained and unrestrained energy minimization calculations using the Discover algorithm (Accelrys, San Diego, CA) and the consistent valence force field [22]. No residue was found in the disallowed region of the Ramachandran plot. The final structures were analyzed using the InsightII program (Accelrys, San Diego, CA). Graphical representations were carried out with the InsightII program. The root-mean-squared-deviation analysis between energy-minimized structures was carried out with the program Molmol [23].

## Results and Discussion

In preliminary tests, the antimicrobial activity of the lead compound *DNal* was tested using the present standard culture conditions. As expected, the peptide did not have substantial antimicrobial activity in broth culture medium (Tables 2 and 3). However, despite these negative results, *DNal* was a valuable basis for development of novel analogues.

**Table 2:** Anti-Candida activity of *DNal* and Gly10 substituted peptides expressed as MIC 90 (µM) at 48 h.

	Peptide													
Candida strain	*DNal*	1	2	3	4	5	6	7	8	9	10	11	12	13
***C.albicans***														
ATCC 76615	>100	>100	>100	>100	>100	>100	>100	51	25	>100	52	>100	>100	>100
ATCC 24433	>100	>100	>100	>100	>100	>100	51	51	25	>100	26	>100	>100	>100
995439	>100	>100	>100	>100	>100	99	51	51	25	>100	26	>100	>100	>100
995147	>100	>100	>100	>100	>100	>100	>100	51	25	>100	26	>100	>100	>100
000954	>100	>100	>100	>100	>100	>100	>100	51	25	>100	52	>100	>100	>100
991185	>100	>100	>100	>100	>100	99	51	51	25	51	52	>100	>100	>100
994199	>100	>100	>100	>100	>100	99	51	51	25	51	26	>100	>100	>100
983201- R1	>100	>100	>100	>100	>100	>100	>100	51	50	>100	52	>100	>100	>100
011587	>100	97	>100	>100	>100	99	>100	51	50	>100	26	>100	>100	>100
***C.glabrata***														
18012	>100	>100	>100	>100	>100	>100	>100	>100	100	>100	>100	>100	>100	>100
995667	>100	>100	>100	>100	>100	>100	>100	>100	100	>100	>100	>100	>100	>100
995651	>100	>100	>100	>100	>100	>100	>100	>100	100	>100	>100	>100	>100	>100
***C.krusei***														
995668	>100	97	>100	>100	>100	99	>100	>100	50	>100	52	>100	>100	>100
991388	>100	97	>100	>100	>100	99	>100	>100	50	>100	26	>100	>100	>100
004490	>100	97	>100	>100	>100	99	>100	51	25	>100	26	>100	>100	>100

Each MIC value is the average of at least three independent experiments.

**Table 3:** Antibacterial activity of selected peptides expressed as MIC 100 (µM) at 18 h.

Peptide						
Bacterial strain	*DNal*	3	4	8	9	10
**Gram-negative bacteria**						
*Acinetobacter b.* ATCC 19606	>100	100	50	12.5	25	12.5
*Escherichia coli* D21	>100	50	100	12.5	50	6.25
*Pseudomonas a.* ATCC 27853	100	50	50	50	25	25
*Pseudomonas syringae* pv tobaci	>100	>100	>100	25	100	25
**Gram-positive bacteria**						
*Bacillus megaterium* Bm 11	6	6.25	6.25	1.56	3.125	3.125
*Staphylococcus a.* ATCC 25923	>100	>100	100	12.5	50	12.5
*Staphylococcus e.* ATCC 12228	50	50	25	6.25	12.5	6.25

Each MIC value is the average of at least three independent experiments.

The Gly10 residue was replaced with several conventional and unconventional amino acids characterized by different degrees of conformational rigidity (Table 1 and Figure S1). Such changes were expected to significantly influence the folding preference of the peptide and, consequently, the antimicrobial activity. The Gly10-substituted peptides were tested against several pathogenic strains of *C. albicans*, *C. glabrata*, and *C. krusei* (Table 2) and against a panel of gram-positive and gram-negative bacteria (Table 3).

The peptides **1** and **2** are Cα,α-disubstituted. Cα-alkylation, and in particular Cα-methylation, by means, for example, of 2-aminoisobutyric acid (Aib), has been widely used to explore the conformational requirements for bioactivity [24]. A further rationale for introduction of Cα,α- disubstituted glycine residues was that the isovaline (α-methyl-α-ethylglycine) residue is present in antifungal peptides such as alamethicin, zervamicin, and antiamboebin [25]. Among Cα,α-disubstituted glycine, Aib-^L^Xxx segments are the preferred option to populate local type-I/III conformations [24]. In addition, the conformational behavior of the cycloaliphatic sub-family of the Cα.α-symmetrically disubstituted Gly residues, generally denoted as Ac_n_c, is closely correlated to that of Aib [26]. Conversely, diethylglycine (Deg) containing peptides have a greater tendency to form extended backbone conformations in strongly interacting solvents such as water [26]. Similarly, the *tert*-butyl glycine (tButGly) also called *tert*-leucine residue, inserted in peptide **3**, prefers extended (β sheet-like) or semiextended [(Pro)_n_-like] conformations [27]. Peptides **1** and **2** containing an Aib and a Deg residue in position 10, respectively, did not show antimicrobial activity. The L-α-t-butylglycine (tButGly) substituted peptide **3** was likewise inactive.

Subsequently, we designed derivatives containing the cyclic achiral dialkylglycine residues 1-amino-cyclopropane carboxylic acid (Ac3c), 1-amino-cyclobutane carboxylic acid (Ac4c), 1-amino-cyclopentane carboxylic acid (Ac5c), and 1-amino-cyclohexane carboxylic acid (Ac6c) (Figure S1) in the same position 10. Compound **4**, in which Gly10 was replaced by Ac3c containing a cyclopropane moiety, was inactive. Conversely, the peptides **5**, **6**, and **7**, in which the size of cycloalkyl moiety of the residues Ac4c, Ac5c, and Ac6c progressively increases, showed a parallel increase in their antimicrobial activity. Indeed, whereas compound **5** showed only weak activity, compounds **6** and **7**, containing the Ac5c and Ac6c residues, respectively, possessed substantial activity.

Based on these results, we designed the compound **8** containing the 2-aminoindane-2-carboxylic acid (Aic) residue that, from a structural point of view, can be considered an aromatic analogue of the Ac5c. Considering the conformational preferences, Aic torsional angles are limited to values around φ = −50°, ψ = −50°, and φ = +50°, ψ = +50° [28]. Compound **8** showed a quite good anti-Candida activity. In addition to the anti-Candida activity, compound **8** effectively inhibited the bacterial strains examined. Therefore, this compound represents a promising agent, marked by both antifungal and antibacterial activity. Because compound **8** can also be considered as a phenylalanine with a constrained side chain, we synthesized an analogue bearing a Phe residue in position 10. However, the resulting compound, peptide **9**, had decreased activity in all biological assays. Therefore, it appears that the conformational constraints imposed by the Aic residue are essential for activity.

Activity was preserved when a L-cyclohexylalanine (Cha) residue was inserted in position 10 (peptide **10**). Cha can be considered an aliphatic analogue of phenylalanine with high tendency to helical formation [29]. This compound exerted both anti-Candida and antibacterial activity with a potency similar to that of peptide **8**.

**Figure 1:**
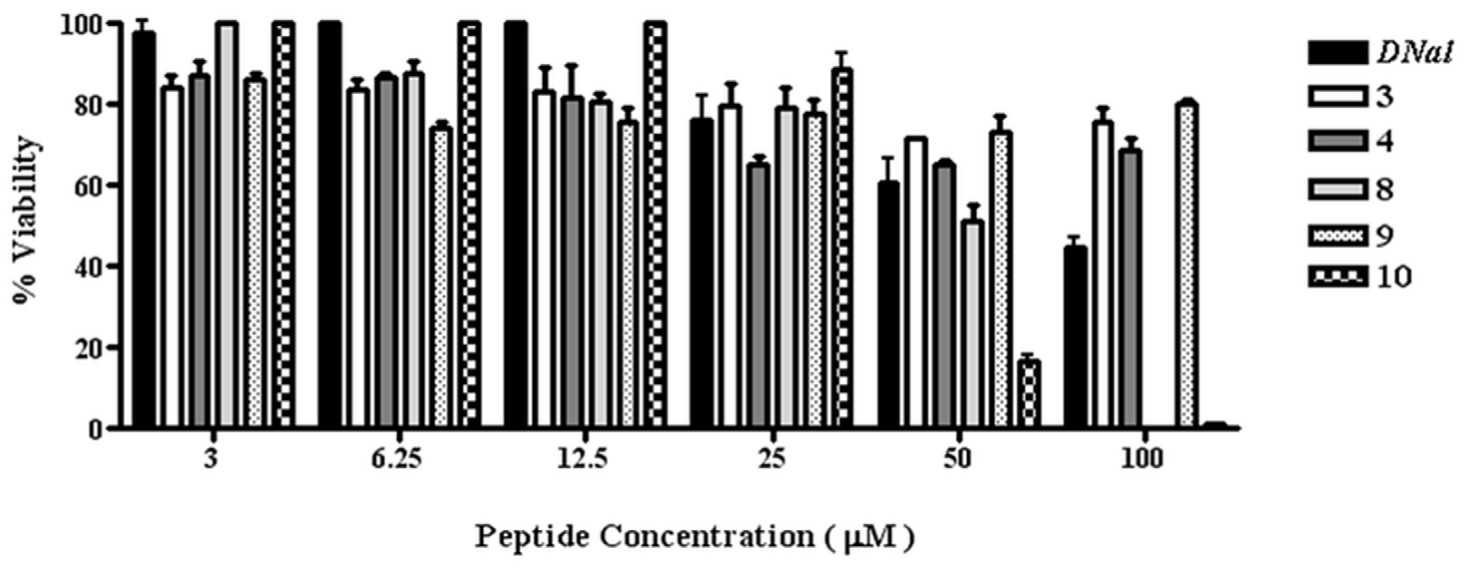
Effects of the synthetic melanocortins on viability of HaCat cells. Cells were plated in wells of a microtiter plate, at 4×10^4^ cells/well in DMEM supplemented with 2% serum without antibiotic. After overnight incubation at 37°C in a 5% CO_2_ atmosphere, the medium was replaced with 100 µl fresh DMEM supplemented with the peptides at different concentrations. After 24 h of peptide treatment, cell viability was determined by the inhibition of MTT reduction to insoluble formazan (see Materials and Methods for additional information). Cell viability is expressed as percentage of viability in control cells (cells not treated with the peptide).

A comprehensive toxicological investigation was not an aim in this paper. Indeed, the objective was definition of structure/activity relationship that could help design of melanocortin-based novel antimicrobials. However, as a preliminary assessment of potential toxicity, peptides *DNal*, **3, 4, 8, 9**, and **10** were tested for their hemolytic activity (Table 4). No significant hemolysis was noted for the peptides *DNal*, **3**, **8**, and **10** up to a concentration of 12.5 µM. A weak activity (<10% hemolysis) was associated with the parent peptide *DNal* up to 100 µM whereascompounds **3**, **8**, and **10** caused moderate lysis of human red blood cells (from 10% to 24–34%) at concentrations from 25 to 100 µM, with a slightly greater effect for compound **10**. Peptides **4** and **9** exerted the greatest hemolytic effect, causing from ∼20% to 30 or 49% cell lysis at concentrations ranging from 25 µM to 100 µM. The potential cytotoxic activity of these compounds was also investigated in nucleated mammalian cells, such as human keratinocytes. As reported in Figure 1, peptide **10** was completely devoid of toxic activity up to a concentration of 12.5 µM, but it exerted toxic effects at 50–100 µM concentrations. Conversely, a moderate toxic effect (less than 20% mortality), was found for the other compounds up to 25 M. At the highest peptide concentration used (100 µM) peptides **3**, **4** and **9** produced less than 30% cell death, whereas a more pronounced toxicity was found for the peptides *DNal* and **8**, that caused approximately 50–100% killing.

**Table 4:** Hemolytic activity^a^ (%) of selected peptides.

Peptide conc. (µM)	1.5	3	6.25	12.5	25	50	100
*DNal*	0±0.2	0±0.9	1±0.6	1±0.5	2±1.06	5±1.5	7±2.1
**3**	5±1.9	5.6±3	6±1.6	7±0.4	15±3.1	16±1	23±0.1
**4**	0±0	2±0.5	4±0.4	10±1	20±2.1	22±0.5	30±0.2
**8**	0.5±1	2±1.7	2±1.1	3±1.7	9±1.9	13±1.8	24±0.4
**9**	4±0.1	6±0.4	9±1.9	14±1.4	24±0.1	33±1.3	49±4.1
**10**	1±0.7	2±1.3	5±0.1	5±0.4	10±1.5	10±0.2	34±1

Values are the mean of three independent experiments ± SD.

In further investigations on structure-activity relationships, Gly10 was replaced with -amino acids. The α-amino acids exist as part of certain natural occurring peptides isolated from prokaryotes and marine organisms. Because of their extra methylene group, β-amino acids are not recognized by mammal proteases and are intrinsically resistant to enzymatic degradation [30]. Further, they show precise conformational preferences. In particular, the conformationally flexible noncoding β-alanine (β-Ala) residue shows two energetically preferred conformations: a folded conformation (μ∼±65±10°) and an extended conformation (μ∼±165±10°) that are easily accommodated in both acyclic and cyclic peptides where they determine the overall molecular structures [31]. The cyclic (1R,2R)-2-aminocyclopentane carboxylic acid (Acpc) is a more constrained -amino acid that determines a so called “12-helix” (a 12-membered ring hydrogen bonds) when inserted into a peptide sequence [32]. Compounds **11** and **12**, containing β-Ala and Acpc in position 10, respectively, were synthesized and examined; both showed no anti-Candida activity.

Finally, we synthesized an analogue of *DNal* in which an additional Gly residue was inserted between the Trp9 and the Lys11 residues (peptide **13**). The aim was to evaluate the effect of increased peptide flexibility on antimicrobial activity; compound **13** showed no activity.

Subsequently, we performed a conformational study using solution NMR on compounds **8** and **10**; the parent peptide *DNal* was investigated for comparison. NMR spectroscopy studies were performed in DPC/SDS 8:2 micelles solution. These micelles were used as rough mimetics of yeast membranes [33]. Complete ^1^H NMR chemical shift assignments were effectively achieved for the peptides according to the Wüthrich procedure [34]. (Tables S3, S4, S5). For both compounds **8** and **10**, upfield shift of the Hα NMR signals, low values of the temperature coefficients of the amide protons, and diagnostic NOEs (Tables S6 and S7) indicated that residues 8–13 are in a helical conformation. Few medium range NOEs between the hydrophobic side chains of residues 7 and 10 were observed in the NOESY spectrum of **10** but not in that of **8** (Tables S6 and S7), suggesting higher conformational stability for the former peptide. Conversely, NMR parameters, including for example lower number of medium range diagnostic NOEs (Table S8), indicate that *DNal* has greater conformational flexibility relative to peptides **8** and **10**.

**Figure 2:**
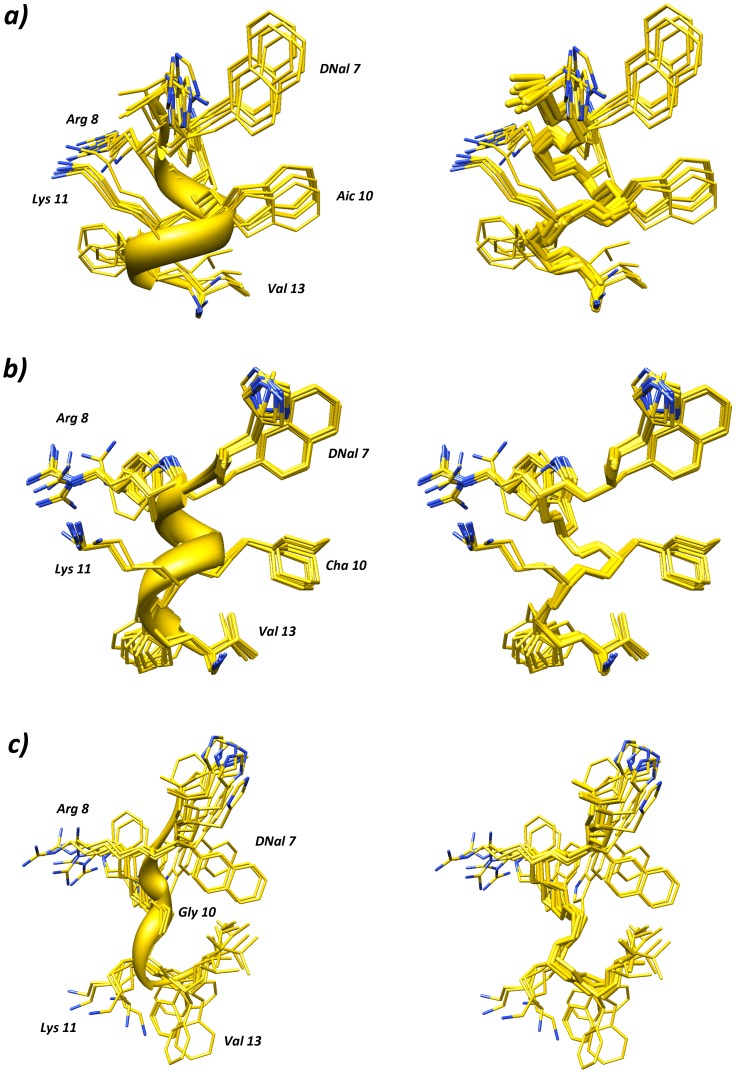
Stereoview of the superimposition of the 10 lowest energy conformers of peptide 8 (a), 10 (b), and ***DNal*** **(c) in SDS/DPC 8:2 solution.** Structures were superimposed using the backbone heavy atoms. Heavy atoms are shown in yellow (except for nitrogen, in blue). To improve clarity, hydrogen atoms are not shown. Backbone atoms of the lowest energy conformer are denoted as a ribbon.

NMR-derived constraints for peptides **8**, **10**, and *DNal* were used as input data for a simulated annealing structure calculation according to the standard protocol of DYANA program [21]. Figures 2a and 2b show the superposition of the best 10 NMR structures of the peptides **8** and **10**, respectively, overlapped at the level of backbone atoms. The bundle reveals a high structural similarity (backbone RMSD≤0.40 Å for peptide **8**, and ≤0.20 Å for peptide **10**), suggesting that NMR structures are defined with high precision.

Analysis of these NMR structures using Molmol [23] identified the prevalence of regular α-helices in the Arg8-Val13 segment of both peptides. Interestingly, the helical structure showed amphipathic distribution of the side chains, with Arg8 and Lys11 side chains on one side of the helix and *D*Nal(2′)7, Aic10 (Cha10), Phe12 and Val13 on the other side (Figure 2a, b).

The *DNal* structure was much more flexible (backbone RMSD = 0.84 Å) and only turn motifs could be detected along its structure. In particular, two β-turns along residues 7–10 and 10–13 (Figure 2c) are consistent with our earlier results [11]. The *DNal* structure is better described as a nascent helix i.e. a helix in equilibrium with disordered structures [35].

Conformational differences could account for the enhanced antimicrobial activity observed in peptides **8** and **10** relative to the parent sequence *DNal*. The amphipathic helical structure along residues 8–13 is likely responsible for the superior antimicrobial activity of the substituted peptides whereas the partial lack of helicity in *DNal* probably contributes to its reduced activity. Indeed, the residues Deg [26], tButGly [27], Ac3c [36], β-Ala [31], and Acpc [32], which do not stabilize regular right handed helices, did not promote activity in the corresponding peptides.Therefore, consistent with previous observations, the amphipathic helical content proved to be a key factor in the activity of antimicrobial peptides [37], [38].

## Conclusions

The present research discovered novel synthetic melanocortins that exert broad-spectrum antimicrobial activity. The main advance of these molecules, relative to the known α-MSH-related compounds, consists of their capacity of killing growing organisms in standard culture conditions. Indeed, previous investigations on antimicrobial melanocortins were not performed in culture media that allow microorganisms to grow. Although, those studies were very helpful to promote discovery of synthetic antimicrobial melanocortins, standard microbiology testing is required for a realistic use in clinical settings. This prerequisite was clearly met by the peptides **8** and **10**, in which Gly10 of *DNal* was replaced by the residues Aic and Cha, respectively. Although these peptides also reduced viability of human cells, these detrimental influences occurred at greater concentrations relative to effective antibacterial concentrations. Overall, the structural preferences associated with antimicrobial activity identified in this research can help further development of synthetic melanocortins with enhanced therapeutic index.

## Supporting Information

Figure S1 Chemical structure of amino acids replacing Gly10 of α-MSH.
(DOC)

Figure S2 Analytical HPLC data of synthesized peptides.
(DOC)

Table S1 Physico-chemical properties of the peptides.
(DOC)

Table S2 Amino acid analysis of the peptides.
(DOC)

Table S3 NMR Resonance Assignments of Peptide **8** in DPC/SDS Solution at 25°C.
(DOC)

Table S4 NMR Resonance Assignments of Peptide **10** in DPC/SDS Solution at 25°C.
(DOC)

Table S5 NMR Resonance Assignments of Peptide *DNal* in DPC/SDS Solution at 25°C.
(DOC)

Table S6 NOE Derived Upper Limit Constraints of Peptide **8** in DPC/SDS Solution at 25°C.
(DOC)

Table S7 NOE Derived Upper Limit Constraints of Peptide **10** in DPC/SDS Solution at 25°C.
(DOC)

Table S8 NOE Derived Upper Limit Constraints of Peptide *DNal* in DPC/SDS Solution at 25°C.
(DOC)
