# Supplementary material for: Novel α-MSH Peptide Analogues with Broad Spectrum Antimicrobial Activity
Source: PLoS One. 2013 Apr 23;8(4):e61614. doi: 10.1371/journal.pone.0061614 (PMC3634028; doi:10.1371/journal.pone.0061614)
Supplement: Figure S1 — Chemical structure of amino acids replacing Gly10 of α-MSH. (DOC) [file pone.0061614.s001.doc]

**Figure S1**. Chemical structure of amino acids replacing Gly10 of α-MSH
