# Supplementary material for: Novel α-MSH Peptide Analogues with Broad Spectrum Antimicrobial Activity
Source: PLoS One. 2013 Apr 23;8(4):e61614. doi: 10.1371/journal.pone.0061614 (PMC3634028; doi:10.1371/journal.pone.0061614)
Supplement: Figure S2 — Analytical HPLC data of synthesized peptides. (DOC) [file pone.0061614.s002.doc]

**Figure S2**. Analytical HPLC data of synthesized peptides^a^


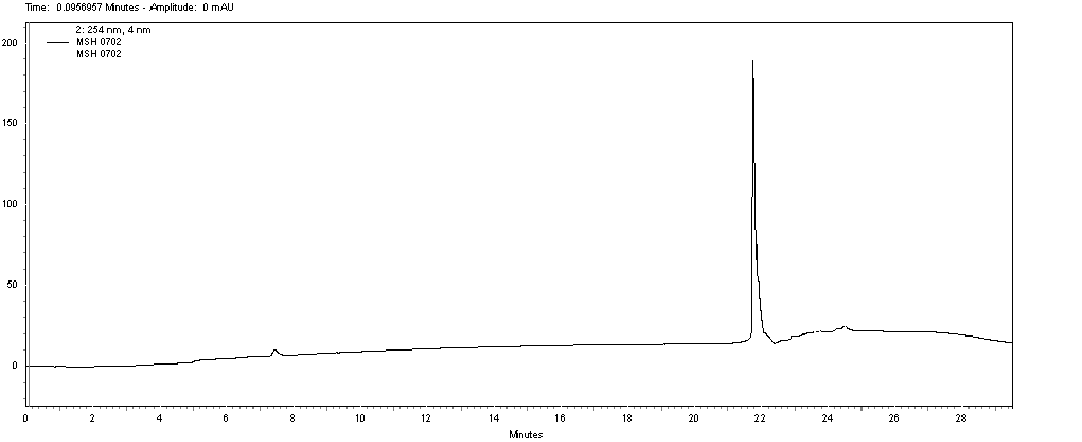


Compound **1**


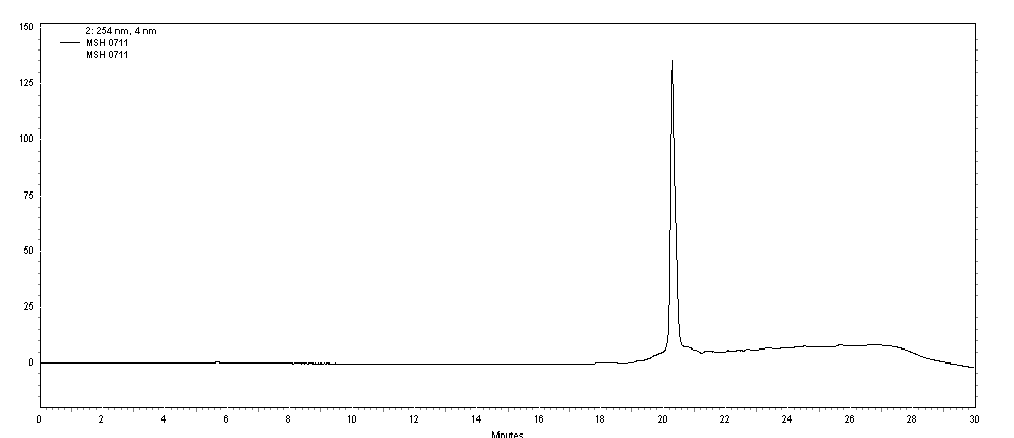


Compound **2**


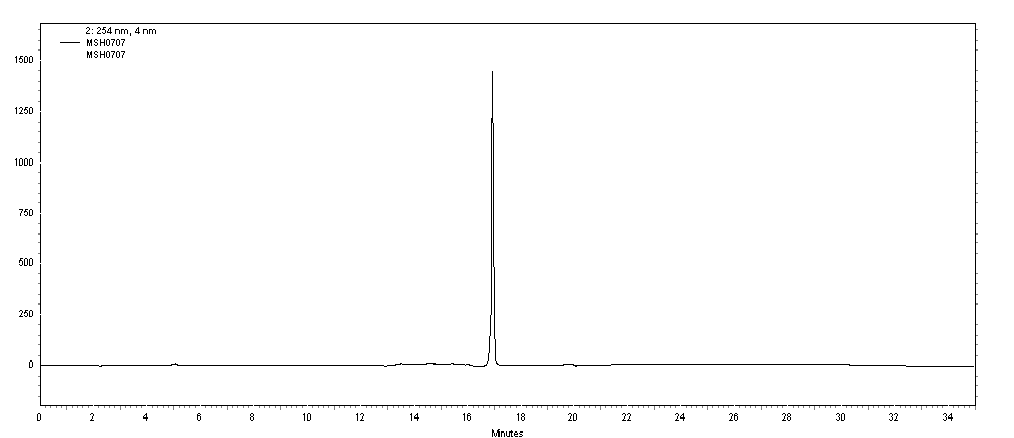


Compound **3**


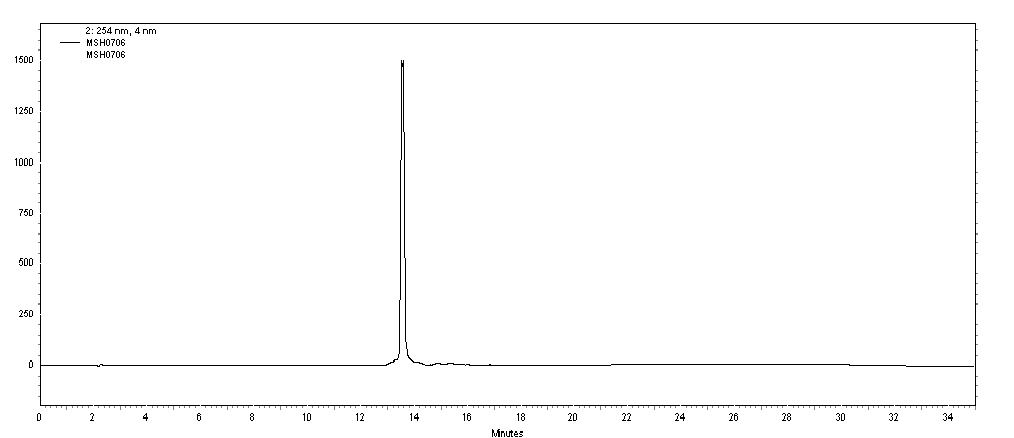


Compound **4**


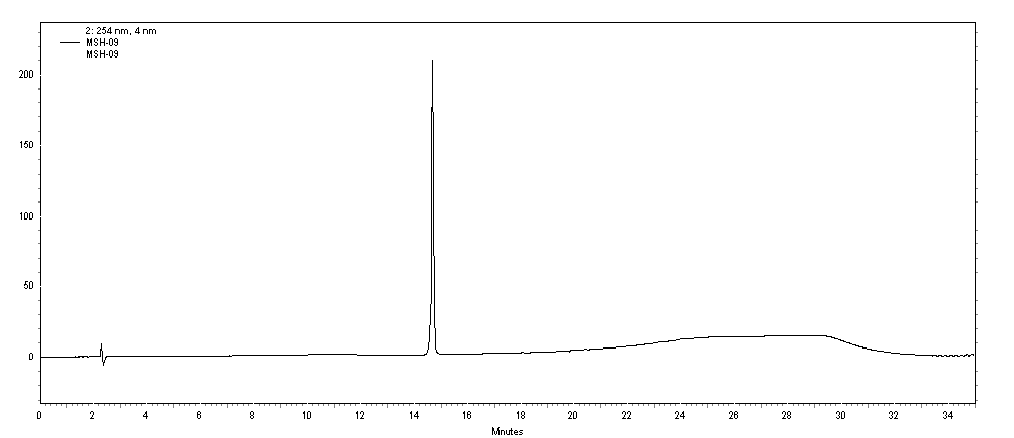


Compound **5**


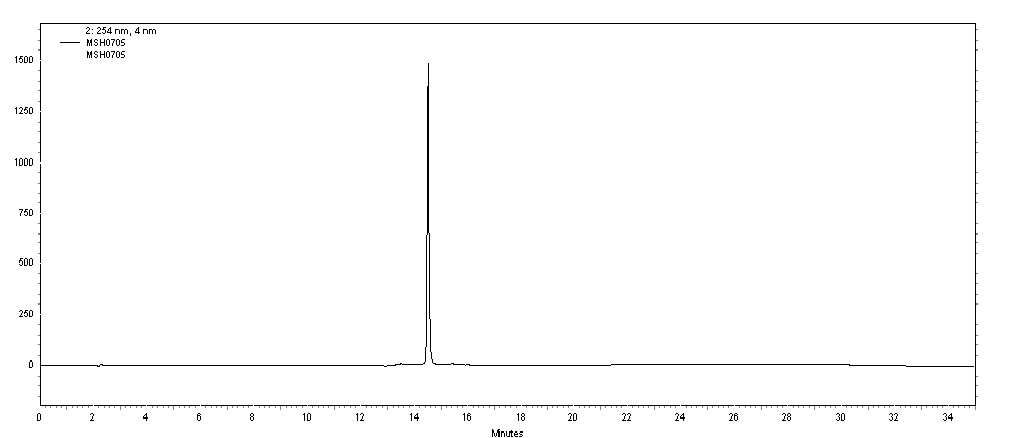


Compound **6**


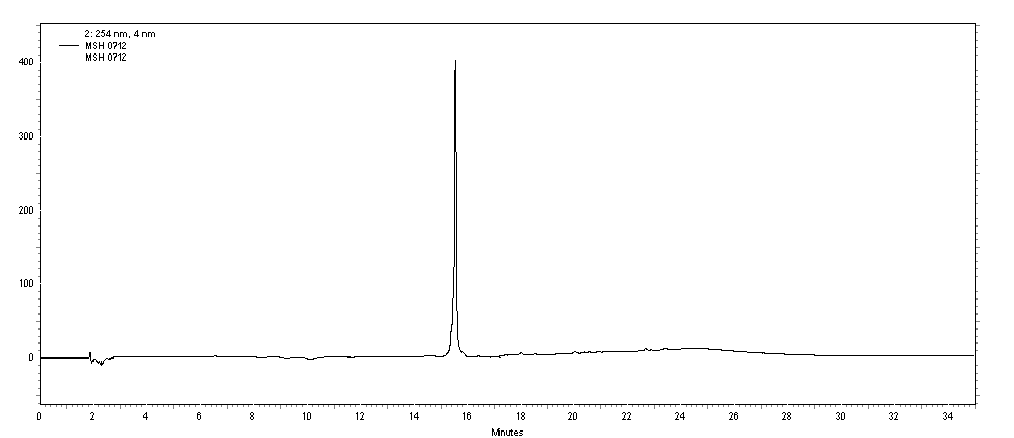


Compound **7**


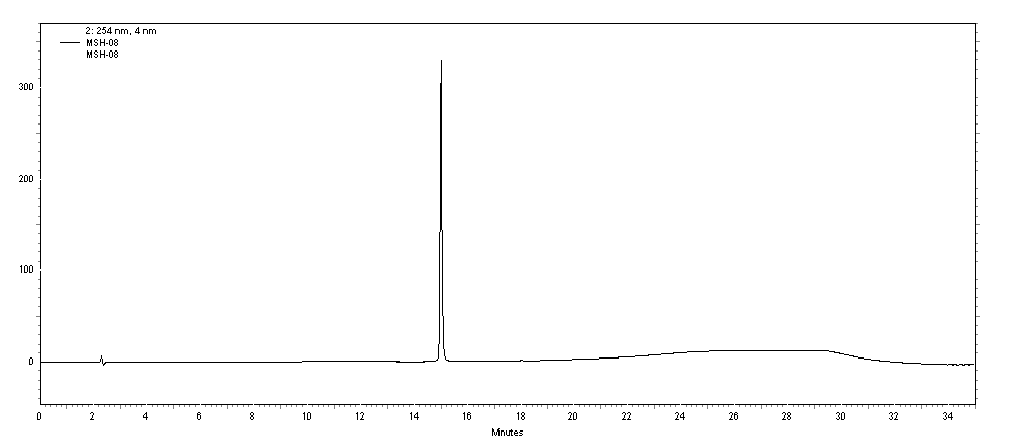


Compound **8**


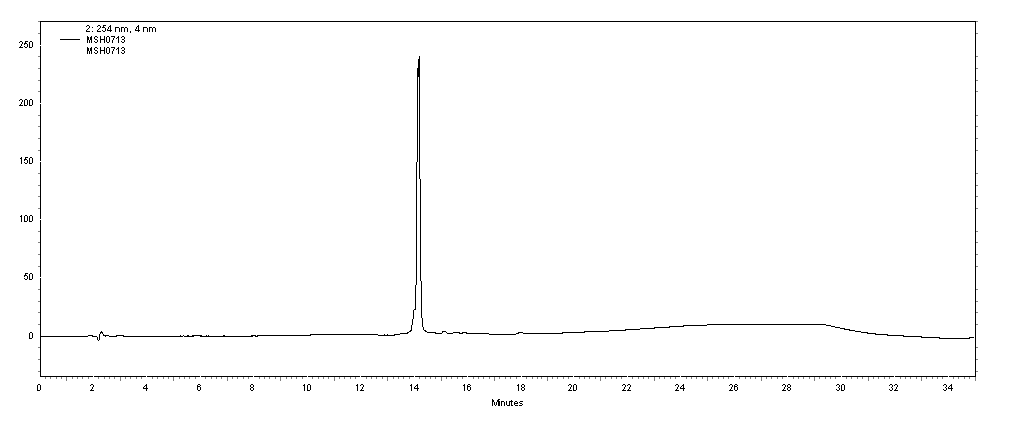


Compound **9**


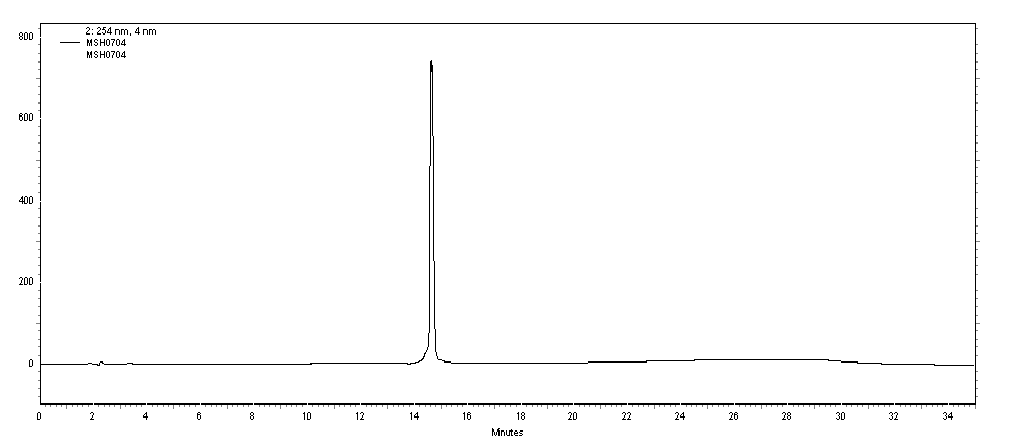


Compound **10**


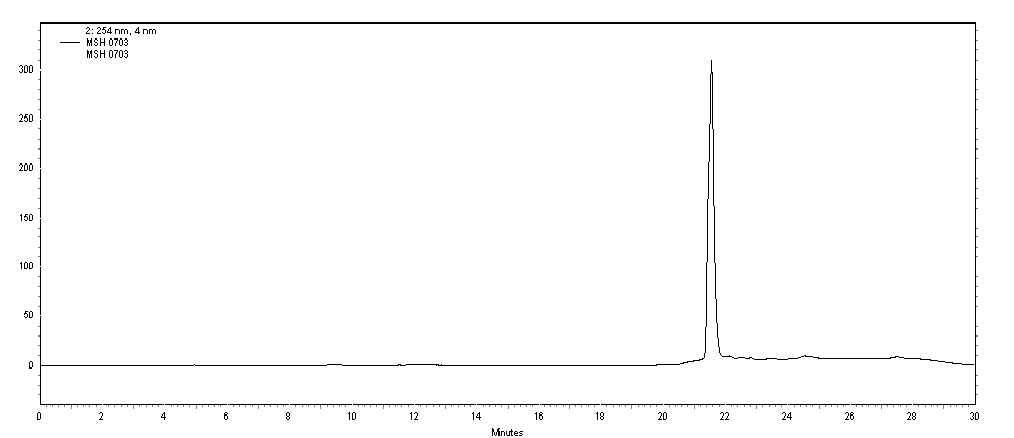


Compound **11**

**
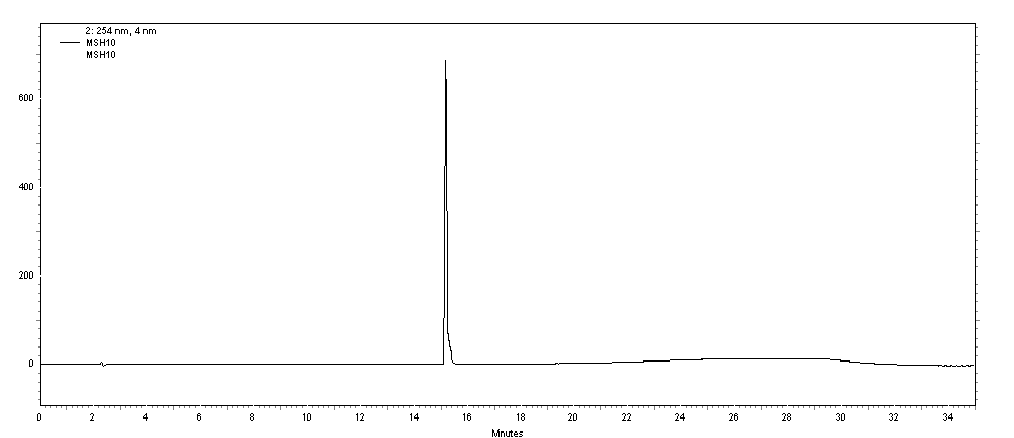
**Compound **12**


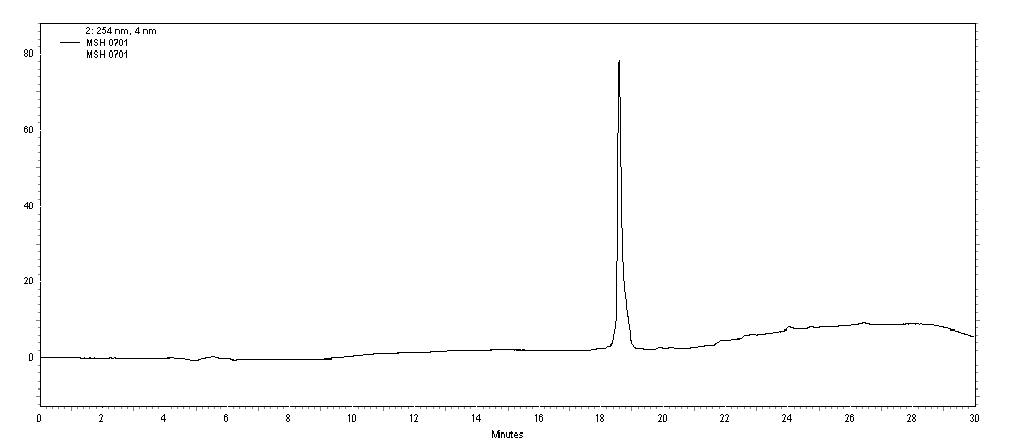


Compound **13**

^a^ Method: The analyses were monitored at 230 and 254 nm and integrated with a Shimadzu diode array detector mod. SPD-M10AVP dual wavelength absorbance detector model UV-D. An analytical Phenomenex Luna C18 column was used with a flow rate of 1 mL/min.
