# Supplementary material for: Novel α-MSH Peptide Analogues with Broad Spectrum Antimicrobial Activity
Source: PLoS One. 2013 Apr 23;8(4):e61614. doi: 10.1371/journal.pone.0061614 (PMC3634028; doi:10.1371/journal.pone.0061614)
Supplement: Table S1 — Physico-chemical properties of the peptides. (DOC) [file pone.0061614.s003.doc]

**Table S1:** Physico-chemical properties of the peptides

| **Pep#** | **R_f_** ^a^ | **M.W.** | **MS,** *m/z* |
| --- | --- | --- | --- |
| **1** | 2.97 | 1182.41 | 1183.90 |
| **2** | 3.31 | 1153.41 | 1154.30 |
| **3** | 3.08 | 1139.38 | 1139.00 |
| **4** | 2.97 | 1221.52 | 1221.80 |
| **5** | 2.44 | 1179.44 | 1179.90 |
| **6** | 2.47 | 1151.39 | 1151.70 |
| **7** | 2.64 | 1181.46 | 1181.60 |
| **8** | 2.79 | 1227.49 | 1227.40 |
| **9** | 3.84 | 1193.47 | 1194.90 |
| **10** | 2.55 | 1181.46 | 1182.60 |
| **11** | 2.75 | 1165.41 | 1165.60 |
| **12** | 2.73 | 1179.44 | 1180.90 |
| **13** | 2.95 | 1215.49 | 1215.30 |

^a^HPLC *k’* = [(peptide retention time – solvent retention time)/solvent retention time] in a solvent system of 10% CH_3_CN in 0.1% TFA and a gradient to 90% CH_3_CN over 20 min. An analytical Phenomenex Luna C18 (2) column was used with a flow rate of 1 mL/min.
