# Supplementary material for: Novel α-MSH Peptide Analogues with Broad Spectrum Antimicrobial Activity
Source: PLoS One. 2013 Apr 23;8(4):e61614. doi: 10.1371/journal.pone.0061614 (PMC3634028; doi:10.1371/journal.pone.0061614)
Supplement: Table S2 — Amino acid analysis of the peptides. (DOC) [file pone.0061614.s004.doc]

**Table S2** Amino acid analysis of the peptides

| **Peptide** | His | DNal | Arg | Trp^a^ | Xaa^b^ | Lys | Phe | Val |
| --- | --- | --- | --- | --- | --- | --- | --- | --- |
| **1** | 0.99 | 0.95 | 0.93 | - | 0.94 | 0.98 | 0.93 | 1.02 |
| **2** | 0.94 | 0.92 | 0.95 | - | 1.01 | 0.91 | 0.98 | 0.93 |
| **3** | 0.98 | 0.98 | 0.90 | - | 0.96 | 0.91 | 0.94 | 0.96 |
| **4** | 0.99 | 1.02 | 0.95 | - | 0.99 | 0.98 | 0.98 | 0.96 |
| **5** | 1.01 | 0.98 | 0.98 | - | 0.97 | 0.90 | 0.92 | 1.03 |
| **6** | 0.98 | 0.95 | 0.99 | - | 0.96 | 1.03 | 1.05 | 0.97 |
| **7** | 0.99 | 0.94 | 0.93 | - | 0.97 | 0.92 | 0.91 | 0.94 |
| **8** | 0.93 | 0.98 | 0.97 | - | 0.99 | 0.89 | 0.91 | 0.94 |
| **9** | 0.92 | 0.91 | 1.03 | - | 1.01 | 0.98 | 0.92 | 0.96 |
| **10** | 1.01 | 0.93 | 0.87 | - | 0.92 | 0.91 | 0.96 | 0.98 |
| **11** | 0.98 | 0.89 | 1.03 | - | 0.97 | 0.92 | 0.97 | 0.93 |
| **12** | 0.95 | 0.95 | 0.96 | - | 0.89 | 1.08 | 0.98 | 0.91 |
| **13** | 0.91 | 0.98 | 0.96 | - | 0.98 | 0.89 | 0.96 | 1.02 |

^a^Trp was not well determined due to decomposition under these conditions. ^b^ Value for the corresponding amino acid (see Table 1).
