# Supplementary material for: Novel α-MSH Peptide Analogues with Broad Spectrum Antimicrobial Activity
Source: PLoS One. 2013 Apr 23;8(4):e61614. doi: 10.1371/journal.pone.0061614 (PMC3634028; doi:10.1371/journal.pone.0061614)
Supplement: Table S3 — NMR Resonance Assignments of Peptide 8 in DPC/SDS Solution at 25°C. (DOC) [file pone.0061614.s005.doc]

**Table S3.** NMR Resonance Assignments^a^ of Peptide **8** in DPC/SDS Solution at 25 °C.

| Residue | NH (exc, -Δδ/ΔT)^b^ | C^α^H | C^β^H | Others |
| --- | --- | --- | --- | --- |
| His^6^ |  | 4.36 | 2.54, 2.90 |  |
| *D*Nal^7^ | 9.11 (f, 8.2) | 4.77 | 3.19, 2.94 | 7.43(δ);7.33(ε);7.75;7.81(ζ) |
| Arg^8^ | 8.12 (f, 7.1) | 3.95 | 1.53, 1.75 | 1.09, 1.29(γ); 2.83; 2.90(δ); 7.02(ε) |
| Trp^9^ | 7.98 (ms, 4.3) | 4.64 | 3.25. 3.34 | 7.34(δ). 7.43,10.14(ε); 6.75,7.33(ζ); 6.98( η) |
| Aic^10^ | 8.13 (f, 6.1) |  | 3.08, 3.25, 3.57. 4.39 | 6.71, 7.12(δ); 6.83,6.90(ε); |
| Lys^11^ | 7.77 (s, 2.3) | 4.08 | 1.22, 1.37 | 1.12, 1.22 (δ),1.63,182 (ε) |
| Phe^12^ | 7.90 (s, 3.0) | 4.45 | 3.26, 3.38 | 7.43(δ); 7.31(ε) |
| Val^13^ | 7.54(s, 3.1) | 3.93 | 2.25 | 1.02,1.13(γ) |

^a^ Obtained at pH = 5, with TSP (δ 0.00 ppm) as reference shift. Chemical shifts are accurate to ±0.02 ppm.

^b^ exc = NH exchange rate (f, fast; ms, moderately slow; s, slow;); -Δδ/ΔT = temperature coefficients (ppb/K) calculated in the range 25-40 °C. Further signals: CONH_2_, 6.96, 7.11 ppm.
