# Supplementary material for: Novel α-MSH Peptide Analogues with Broad Spectrum Antimicrobial Activity
Source: PLoS One. 2013 Apr 23;8(4):e61614. doi: 10.1371/journal.pone.0061614 (PMC3634028; doi:10.1371/journal.pone.0061614)
Supplement: Table S4 — NMR Resonance Assignments of Peptide 10 in DPC/SDS Solution at 25°C. (DOC) [file pone.0061614.s006.doc]

**Table S4.** NMR Resonance Assignments^a^ of Peptide **10** in DPC/SDS Solution at 25°C.

| Residue | NH (exc, -Δδ/ΔT)^b^ | C^α^H | | C^β^H | Others | |  |
| --- | --- | --- | --- | --- | --- | --- | --- |
| His^6^ |  | 4.39 | 2.54, 2.90 | | |  | |
| *D*Nal^7^ | 9.22 (f, 7.8) | 4.76 | 3.13, 3.40 | | | 7.69,7.87(δ);7.79,7.85(ε);7.31;7.73(ζ) | |
| Arg^8^ | 8.16 (f, 6.6) | 3.87 | 1.54, 1.78 | | | 1.05, 1.26(γ); 2.81; 2.88(δ); 7.00(ε) | |
| Trp^9^ | 8.15 (ms, 3.7) | 4.67 | 3.36. 3.43 | | | 7.47(δ). 7.47,10.20(ε); 6.77,7.34(ζ); 6.99( η) | |
| Cha^10^ | 7.61 (f, 5.9) | 4.19 | 1.49,1.67 | | | 0.73, 0.98(δ) | |
| Lys^11^ | 7.69 (s, 2.1) | 4.00 | 1.67, 1.76 | | | 1.24, 1.32 (γ),1.60(δ); 2.87(ε)7.60(ζ); | |
| Phe^12^ | 7.69 (s, 2.2) | 4.40 | 3.18, 3.32 | | | 7.38(δ); 7.30(ε) | |
| Val^13^ | 7.47 (s, 3.2) | 3.86 | 2.17 | | | 0.95, 0.99(γ) | |

^a^ Obtained at pH = 5, with TSP (δ 0.00 ppm) as reference shift. Chemical shifts are accurate to ±0.02 ppm.

^b^ exc = NH exchange rate (f, fast; ms, moderately slow; s, slow;); -Δδ/ΔT = temperature coefficients (ppb/K) calculated in the range 25-40 °C. Further signals: CONH_2_, 6.94, 7.10 ppm.
