# Supplementary material for: Novel α-MSH Peptide Analogues with Broad Spectrum Antimicrobial Activity
Source: PLoS One. 2013 Apr 23;8(4):e61614. doi: 10.1371/journal.pone.0061614 (PMC3634028; doi:10.1371/journal.pone.0061614)
Supplement: Table S5 — NMR Resonance Assignments of Peptide DNal in DPC/SDS Solution at 25°C. (DOC) [file pone.0061614.s007.doc]

**Table S5.** NMR Resonance Assignments^a^ of Peptide *DNal* in DPC/SDS Solution at 25°C.

| Residue | NH (exc, -Δδ/ΔT)^b^ | C^α^H | | C^β^H | | Others | |
| --- | --- | --- | --- | --- | --- | --- | --- |
| His^6^ |  | 4.37 | 2.95, 3.03 | | 6.90(δ);8.31(ε) | |  |
| *D*Nal^7^ | 9.24 (f, 7.7) | 4.65 | 2.96,3.15 | | 7.44,7.73(δ); 7.78(ε) | |  |
| Arg^8^ | 7.74 (f, 6.6) | 4.08 | 1.27, 1.36 | | 0.83,0.87(γ); 2.70; 2.76(δ); 7.05(ε); 6.82(ζ) | |  |
| Trp^9^ | 7.83 (ms, 4.4) | 4.73 | 3.34 | | 7.29(δ). 7.68,10.53(ε); 6.97,7.41(ζ); 7.01( η) | |  |
| Gly^10^ | 8.27 (f, 7.2) | 3.85,3.88 |  | |  | |  |
| Lys^11^ | 7.91 (f, 7.4) | 4.16 | 1.58 | | 1.16 (γ),1.55(δ); 2.84(ε)7.39(ζ); | |  |
| Phe^12^ | 8.08 (f, 5.1) | 4.61 | 3.04, 3.16 | | 7.26(δ); 7.19(ε) | |  |
| Val^13^ | 7.61(ms, 4.9) | 4.06 | 2.07 | | 0.89(γ) | |  |

^a^ Obtained at pH = 5, with TSP (δ 0.00 ppm) as reference shift. Chemical shifts are accurate to ±0.02 ppm.

^b^ exc = NH exchange rate (f, fast; ms, moderately slow; s, slow;); -Δδ/ΔT = temperature coefficients (ppb/K) calculated in the range 25-40 °C. Further signals: CONH_2_, 7.04, 7.35 ppm.
