# Supplementary material for: Novel α-MSH Peptide Analogues with Broad Spectrum Antimicrobial Activity
Source: PLoS One. 2013 Apr 23;8(4):e61614. doi: 10.1371/journal.pone.0061614 (PMC3634028; doi:10.1371/journal.pone.0061614)
Supplement: Table S6 — NOE Derived Upper Limit Constraints of Peptide 8 in DPC/SDS Solution at 25°C. (DOC) [file pone.0061614.s008.doc]

**Table S6.** NOE Derived Upper Limit Constraints of Peptide **8** in DPC /SDS Solution at 25°C.

6 HIS HA 6 HIS HB2 2.83

6 HIS HA 6 HIS HB3 2.83

6 HIS HA 7 DNAL HN 2.77

6 HIS HA 8 ARG HN 4.62

6 HIS HB2 7 DNAL HN 3.70

6 HIS HB3 7 DNAL HN 3.70

7 DNAL HN 7 DNAL QB 3.95

7 DNAL HN 7 DNAL HD1 5.31

7 DNAL HN 7 DNAL HD2 3.73

7 DNAL HN 8 ARG HN 3.45

7 DNAL HA 7 DNAL HD1 2.74

7 DNAL HA 8 ARG HN 3.39

7 DNAL HB2 7 DNAL HD1 3.64

7 DNAL HB3 7 DNAL HD1 3.64

7 DNAL QB 7 DNAL HD1 3.27

7 DNAL QB 7 DNAL HD2 3.21

8 ARG HN 8 ARG HA 2.86

8 ARG HN 8 ARG HB2 2.96

8 ARG HN 8 ARG HB3 2.96

8 ARG HN 8 ARG HG2 5.50

8 ARG HN 8 ARG HG3 5.50

8 ARG HN 8 ARG QG 5.17

8 ARG HN 9 TRP HN 2.68

8 ARG HA 8 ARG HB2 2.80

8 ARG HA 8 ARG HB3 2.80

8 ARG HA 8 ARG QB 2.60

8 ARG HA 8 ARG HG2 3.64

8 ARG HA 8 ARG HG3 3.64

8 ARG HA 8 ARG QG 3.28

8 ARG HA 9 TRP HN 3.36

8 ARG HA 11 LYS QB 4.58

8 ARG HB2 9 TRP HN 3.89

8 ARG HB2 9 TRP HD1 5.13

8 ARG HB3 9 TRP HN 3.89

8 ARG HB3 9 TRP HD1 5.13

8 ARG QB 9 TRP HN 3.56

8 ARG QB 9 TRP HD1 4.61

8 ARG HG2 9 TRP HD1 5.16

8 ARG HG3 9 TRP HD1 5.16

8 ARG QG 9 TRP HN 6.38

8 ARG QG 9 TRP HD1 4.64

8 ARG HD2 9 TRP HD1 4.17

8 ARG HD3 9 TRP HD1 4.17

8 ARG QD 9 TRP HD1 4.00

9 TRP HN 9 TRP HB2 3.05

9 TRP HN 9 TRP HB3 3.05

9 TRP HN 9 TRP HE1 5.50

9 TRP HN 10 AIC HN 3.27

9 TRP HA 9 TRP HD1 3.33

9 TRP HA 11 LYS HN 4.89

9 TRP HA 12 PHE HN 3.73

9 TRP HA 12 PHE HB3 3.76

9 TRP QB 10 AIC HN 4.55

10 AIC HN 11 LYS HN 3.30

10 AIC QB2 13 VAL HN 5.45

10 AIC QB2 13 VAL HB 6.04

10 AIC QB2 13 VAL QQG 6.65

11 LYS HN 11 LYS HA 2.83

11 LYS HN 11 LYS QB 3.83

11 LYS HN 11 LYS HG2 4.04

11 LYS HN 11 LYS HG3 4.04

11 LYS HN 11 LYS QG 3.85

11 LYS HA 11 LYS HG2 4.14

11 LYS HA 11 LYS HG3 4.14

11 LYS HA 11 LYS QG 3.76

11 LYS HA 12 PHE HN 3.14

11 LYS HA 13 VAL HN 3.92

11 LYS HA CNH2 HN1 4.94

11 LYS QB 12 PHE HN 4.67

11 LYS HG2 12 PHE HN 5.50

11 LYS HG3 12 PHE HN 5.50

12 PHE HN 12 PHE HB2 2.68

12 PHE HN 12 PHE HB3 2.93

12 PHE HN 13 VAL HN 2.80

12 PHE HA 12 PHE HB2 2.80

12 PHE HA 12 PHE HB3 2.83

12 PHE HA 13 VAL HN 3.61

12 PHE HB2 13 VAL HN 3.45

12 PHE HB3 13 VAL HN 3.48

13 VAL HN 13 VAL HA 2.90

13 VAL HN 13 VAL HB 3.70

13 VAL HN 13 VAL QQG 4.32

13 VAL HN CNH2 HN1 3.21

13 VAL HA 13 VAL HB 2.77

13 VAL HA 13 VAL QG1 3.77

13 VAL HA 13 VAL QG2 3.77

13 VAL HB CNH2 HN1 4.29

13 VAL HB CNH2 HN2 5.25

*CNH2: C-terminal amide.*
