# Supplementary material for: Novel α-MSH Peptide Analogues with Broad Spectrum Antimicrobial Activity
Source: PLoS One. 2013 Apr 23;8(4):e61614. doi: 10.1371/journal.pone.0061614 (PMC3634028; doi:10.1371/journal.pone.0061614)
Supplement: Table S7 — NOE Derived Upper Limit Constraints of Peptide 10 in DPC/SDS Solution at 25°C. (DOC) [file pone.0061614.s009.doc]

**Table S7.** NOE Derived Upper Limit Constraints of Peptide **10** in DPC/SDS Solution at 25°C.

6 HIS HA 7 DNAL HN 2.83

6 HIS HA 7 DNAL HD2 5.50

6 HIS HA 8 ARG HN 4.48

6 HIS HB2 7 DNAL HN 5.50

6 HIS HB2 7 DNAL HD1 5.50

6 HIS HB3 7 DNAL HN 5.50

6 HIS HB3 7 DNAL HD1 5.50

6 HIS QB 7 DNAL HN 4.80

6 HIS QB 7 DNAL HD1 5.28

6 HIS QB 7 DNAL HD2 6.38

7 DNAL HN 7 DNAL HD1 5.50

7 DNAL HN 7 DNAL HE3 4.26

7 DNAL HN 7 DNAL HD2 5.50

7 DNAL HN 8 ARG HN 3.55

7 DNAL HA 7 DNAL HD1 2.96

7 DNAL HA 7 DNAL HD2 3.92

7 DNAL HA 8 ARG HN 3.61

7 DNAL HA 9 TRP HN 5.13

7 DNAL HB2 7 DNAL HD1 3.82

7 DNAL HB3 7 DNAL HD1 3.82

7 DNAL QB 7 DNAL HD1 3.45

7 DNAL HD1 10 CHA HB2 4.88

7 DNAL HD1 10 CHA HB3 4.88

7 DNAL HD1 10 CHA QB 4.59

8 ARG HN 8 ARG HB2 2.86

8 ARG HN 8 ARG HB3 2.86

8 ARG HN 8 ARG HG2 5.50

8 ARG HN 8 ARG HG3 5.50

8 ARG HN 8 ARG HD2 5.50

8 ARG HN 8 ARG HD3 5.50

8 ARG HA 8 ARG HB2 2.71

8 ARG HA 8 ARG HB3 2.90

8 ARG HA 8 ARG HG2 4.07

8 ARG HA 8 ARG HG3 4.07

8 ARG HA 8 ARG QG 3.83

8 ARG HA 8 ARG HD2 5.50

8 ARG HA 8 ARG HD3 5.50

8 ARG HA 8 ARG QD 5.28

8 ARG HA 9 TRP HN 3.58

8 ARG HA 10 CHA HN 4.88

8 ARG HA 11 LYS HN 4.29

8 ARG HB2 8 ARG QG 2.72

8 ARG HB3 8 ARG QD 4.02

8 ARG HB3 9 TRP HD1 4.04

8 ARG HD2 9 TRP HD1 5.50

8 ARG HD3 9 TRP HD1 5.50

9 TRP HN 9 TRP HD1 3.24

9 TRP HN 10 CHA HN 3.27

9 TRP HA 11 LYS HN 3.55

9 TRP HB2 10 CHA HN 4.00

9 TRP HB3 10 CHA HN 4.00

9 TRP QB 10 CHA HN 3.80

9 TRP QB 12 PHE HN 5.73

9 TRP QB 12 PHE QD 8.50

9 TRP HD1 10 CHA QB 4.74

10 CHA HN 10 CHA HB2 2.86

10 CHA HN 10 CHA HB3 2.86

10 CHA HN 11 LYS QD 5.85

10 CHA HA 10 CHA HB2 2.95

10 CHA HA 10 CHA HB3 2.95

10 CHA HA 10 CHA QB 2.72

10 CHA HA 10 CHA QD1 4.93

10 CHA HA 10 CHA QD2 5.15

10 CHA HA 11 LYS HN 3.36

10 CHA HA 13 VAL HN 3.55

10 CHA HA 13 VAL HB 5.31

10 CHA HB2 11 LYS HN 3.79

10 CHA HB3 11 LYS HN 3.79

10 CHA QB 11 LYS HN 3.46

10 CHA QB 13 VAL HN 5.35

10 CHA QB 13 VAL HB 5.94

10 CHA QB 13 VAL QQG 6.55

10 CHA QD2 11 LYS HN 6.39

11 LYS HN 11 LYS HA 2.83

11 LYS HN 11 LYS QB 3.71

11 LYS HN 11 LYS HG2 4.35

11 LYS HN 11 LYS HG3 4.35

11 LYS HN 11 LYS QG 4.15

11 LYS HA 11 LYS QD 5.82

11 LYS HA 13 VAL HN 4.29

11 LYS HA CNH2 HN1 5.50

12 PHE HN 12 PHE HB2 2.99

12 PHE HN 12 PHE HB3 2.99

12 PHE HN 12 PHE QB 2.78

12 PHE HN 13 VAL HN 2.59

12 PHE HN CNH2 HN1 5.50

12 PHE HA 12 PHE HB2 2.90

12 PHE HA 12 PHE HB3 2.90

12 PHE HA 13 VAL HN 3.48

12 PHE HB2 13 VAL HN 3.42

12 PHE HB3 13 VAL HN 3.42

12 PHE QB 13 VAL HN 3.23

12 PHE QD 13 VAL HA 7.62

12 PHE QD 13 VAL QG1 6.50

12 PHE QD 13 VAL QG2 6.50

12 PHE QE 13 VAL HA 5.20

12 PHE QE 13 VAL QG1 6.95

12 PHE QE 13 VAL QG2 6.95

13 VAL HN 13 VAL HB 3.05

13 VAL HN 13 VAL QQG 4.23

13 VAL HN CNH2 HN1 3.58

13 VAL HN CNH2 HN2 4.04

13 VAL HB CNH2 HN1 5.04

13 VAL QG1 CNH2 HN1 5.02

13 VAL QG2 CNH2 HN1 5.02

*CNH2: C-terminal amide.*
