# Supplementary material for: Novel α-MSH Peptide Analogues with Broad Spectrum Antimicrobial Activity
Source: PLoS One. 2013 Apr 23;8(4):e61614. doi: 10.1371/journal.pone.0061614 (PMC3634028; doi:10.1371/journal.pone.0061614)
Supplement: Table S8 — NOE Derived Upper Limit Constraints of Peptide DNal in DPC/SDS Solution at 25°C. (DOC) [file pone.0061614.s010.doc]

**Table S8.** NOE Derived Upper Limit Constraints of Peptide *DNal* in DPC/SDS Solution at 25°C.

6 HIS HA 6 HIS HB2 2.83

6 HIS HA 6 HIS HB3 2.83

6 HIS HA 6 HIS HD2 5.50

6 HIS HA 7 DNAL HN 3.14

6 HIS HB2 6 HIS HD2 3.83

6 HIS HB3 6 HIS HD2 3.83

6 HIS QB 6 HIS HD2 3.52

6 HIS QB 7 DNAL HN 4.58

6 HIS QB 7 DNAL HD2 5.02

7 DNAL HN 7 DNAL QB 3.85

7 DNAL HN 7 DNAL HD1 5.50

7 DNAL HN 7 DNAL HD2 5.07

7 DNAL HA 7 DNAL HD1 3.11

7 DNAL HA 8 ARG HN 3.14

7 DNAL HA 9 TRP HN 5.16

7 DNAL HB2 7 DNAL HD1 3.55

7 DNAL HB2 7 DNAL HD2 2.77

7 DNAL HB2 8 ARG HN 5.50

7 DNAL HB3 7 DNAL HD1 3.55

7 DNAL HB3 7 DNAL HD2 2.77

7 DNAL HB3 8 ARG HN 5.50

7 DNAL QB 7 DNAL HD1 3.38

8 ARG HN 8 ARG HG2 5.50

8 ARG HN 8 ARG HG3 5.50

8 ARG HA 8 ARG HB2 2.96

8 ARG HA 8 ARG HB3 2.96

8 ARG HA 8 ARG HG2 3.52

8 ARG HA 8 ARG HG3 3.52

8 ARG HA 8 ARG QG 3.04

8 ARG HA 8 ARG HD2 5.50

8 ARG HA 8 ARG HD3 5.50

8 ARG HA 9 TRP HN 2.71

8 ARG HA 10 GLY HN 5.10

8 ARG HB2 9 TRP HN 4.04

8 ARG HB3 9 TRP HN 4.04

8 ARG QB 9 TRP HN 3.88

8 ARG HG2 9 TRP HN 5.50

8 ARG HG3 9 TRP HN 5.50

8 ARG QG 8 ARG HE 3.89

8 ARG QG 9 TRP HN 4.78

9 TRP HN 9 TRP HE3 3.70

9 TRP HN 10 GLY HN 3.27

9 TRP HA 10 GLY HN 3.24

9 TRP QB 10 GLY HN 4.39

10 GLY HN 10 GLY HA1 2.55

10 GLY HN 10 GLY HA2 2.55

10 GLY HN 11 LYS HN 3.14

10 GLY HA1 11 LYS HN 2.90

10 GLY HA1 12 PHE HN 5.16

10 GLY HA2 11 LYS HN 2.90

10 GLY HA2 12 PHE HN 5.16

10 GLY QA 12 PHE HN 4.43

11 LYS HN 11 LYS HA 2.80

11 LYS HN 11 LYS QB 3.68

11 LYS HN 11 LYS QG 4.92

11 LYS HN 12 PHE HN 2.96

11 LYS HN 12 PHE QD 7.62

11 LYS HA 11 LYS QD 4.33

11 LYS HA 12 PHE HN 2.77

11 LYS HA 12 PHE QD 7.62

11 LYS HA 13 VAL HN 4.48

11 LYS QB 12 PHE HN 4.11

12 PHE HN 12 PHE HB2 2.96

12 PHE HN 12 PHE HB3 2.96

12 PHE HN 12 PHE QB 2.74

12 PHE HN 13 VAL HN 2.86

12 PHE HA 12 PHE HB2 2.62

12 PHE HA 12 PHE HB3 2.62

12 PHE HA 12 PHE QB 2.40

12 PHE HA 13 VAL HN 2.86

12 PHE HB2 13 VAL HN 3.21

12 PHE HB2 13 VAL QQG 7.62

12 PHE HB3 13 VAL HN 3.21

12 PHE HB3 13 VAL QQG 7.62

12 PHE QD 13 VAL HN 5.85

12 PHE QD 13 VAL QQG 9.50

13 VAL HN 13 VAL HB 2.86

13 VAL HN CNH2 HN1 3.27

13 VAL HA 13 VAL HB 2.83

13 VAL HA CNH2 HN1 3.14

13 VAL HB CNH2 HN1 4.04

13 VAL QQG CNH2 HN1 6.35

*CNH2: C-terminal amide.*
